# Supplementary material for: Suicidal thoughts and behaviours in Cape Town: A cross-sectional study of prevalence and social, contextual, and clinical correlates
Source: SSM Ment Health. Author manuscript; Available in PMC 2026 Aug 7. (PMC13441158; doi:10.1016/j.ssmmh.2026.100638)
Supplement: Supplementary file 1 [file NIHMS2197166-supplement-Figure_S1.docx]

**Figure S1: Co-occurrence of high perceived stress, violence exposure, and mental disorders**

Panels: A) participants without suicidal ideation in the past 30 days, B) participants with suicidal ideation in the past 30 days. Areas represent approximate overlaps derived from Euler fits. Numbers indicate the number of participants in each intersection. In total, 527 participants without ideation and 86 participants with ideation were included. Mental disorder denotes the presence of any assessed current mental disorder. Violence exposure refers to lifetime exposure to violence in the family or community. High stress is defined as a Perceived Stress Score (PSS-4) ≥11. No risk factor indicates absence of all three risk factors. The intersection high stress ∩ violence exposure without mental disorder (n = 2) among participants with ideation could not be represented by the Euler fit and is therefore not shown in panel B.
