# Supplementary material for: Suicidal thoughts and behaviours in Cape Town: A cross-sectional study of prevalence and social, contextual, and clinical correlates
Source: SSM Ment Health. Author manuscript; Available in PMC 2026 Aug 7. (PMC13441158; doi:10.1016/j.ssmmh.2026.100638)
Supplement: Supplementary file 2 [file NIHMS2197166-supplement-Figure_S2.docx]

**Figure S2: Associations between HIV status and past 30-day suicidal thoughts and behaviours**

Adjusted odds ratios (ORs) for associations between HIV status and past 30-day suicidal ideation (black) and past 30-days suicidal thoughts and behaviours (grey). Models adjust for age group, sex, and population group. Error bars indicate 95% confidence intervals (CIs). Suicidal ideation is a binary outcome modelled with logistic regression. Suicidal thoughts and behaviours are coded hierarchically (none, ideation, plan, attempt), with participants assigned to the highest level endorsed, and modelled with ordinal logistic regression.

**Figure S3: Sociodemographic factors and violence exposure associated with past 30-day suicidal thoughts and behaviours**

Adjusted odds ratios (ORs) for associations between sociodemographic factors and violence exposure and past 30-day suicidal ideation (black) and past 30-days suicidal thoughts and behaviours (grey). Models included all sociodemographic factors shown, population group, and HIV status. Error bars indicate 95% confidence intervals (CIs). Suicidal ideation is a binary outcome modelled with logistic regression. Suicidal thoughts and behaviours are coded hierarchically (none, ideation, plan, attempt), with participants assigned to the highest level endorsed, and modelled with ordinal logistic regression.

**Figure S4: Associations between perceived stress and past 30-day suicidal thoughts and behaviours**

Adjusted odds ratios (ORs) for associations between perceived stress and past 30-day suicidal ideation (black) and past 30-days suicidal thoughts and behaviours (grey). Models adjusted for age group, sex, population group, and HIV status. Error bars indicate 95% confidence intervals (CIs). Suicidal ideation is a binary outcome modelled with logistic regression. Suicidal thoughts and behaviours are coded hierarchically (none, ideation, plan, attempt), with participants assigned to the highest level endorsed, and modelled with ordinal logistic regression.

**Figure S5: Associations between specific mental disorders and past 30-day suicidal thoughts and behaviours**

Adjusted odds ratios (ORs) for associations between any current mental disorder and past 30-day suicidal ideation (black) and past 30-days suicidal thoughts and behaviours (grey). Models adjusted for age group, sex, population group, and HIV status. Error bars indicate 95% confidence intervals (CIs). Suicidal ideation is a binary outcome modelled with logistic regression. Suicidal thoughts and behaviours are coded hierarchically (none, ideation, plan, attempt), with participants assigned to the highest level endorsed, and modelled with ordinal logistic regression.

**Figure S6. Average marginal effect of any current mental disorder on past 30-day suicidal ideation**

Average marginal effect (AME), expressed as a percentage-point difference, for the association between any current mental disorder and past 30-day suicidal ideation. Estimates derive from an adjusted logistic regression model including age group, sex, population group, and HIV status. The AME represents the average discrete change in the predicted probability of suicidal ideation comparing participants with any current mental disorder versus none, averaged over the observed covariate distribution. Error bars indicate 95% confidence intervals. The corresponding adjusted odds ratio are presented in Figure S7.

**Figure S7: Associations between any mental disorder and past 30-day suicidal thoughts and behaviours**

Adjusted odds ratios (ORs) for associations between any current mental disorder and past 30-day suicidal ideation (black) and past 30-days suicidal thoughts and behaviours (grey). Models adjusted for age group, sex, population group, and HIV status. Error bars indicate 95% confidence intervals (CIs). Suicidal ideation is a binary outcome modelled with logistic regression. Suicidal thoughts and behaviours are coded hierarchically (none, ideation, plan, attempt), with participants assigned to the highest level endorsed, and modelled with ordinal logistic regression.

**Figure S8. Average marginal effect of the number of current mental disorder on past 30-day suicidal ideation**

Average marginal effects (AMEs), expressed as percentage-point differences, for the association between the number of current mental disorders and past 30-day suicidal ideation. Estimates derive from an adjusted logistic regression model including age group, sex, population group, and HIV status. AMEs represent the average discrete change in the predicted probability of suicidal ideation for each category of number of current mental disorders (1, 2, or ≥3) compared with none, averaged over the observed covariate distribution. Error bars indicate 95% confidence intervals. The corresponding adjusted odds ratio are presented in Figure S9.

**Figure S9: Associations between number of mental disorders and past 30-day suicidal thoughts and behaviours**

Adjusted odds ratios (ORs) for associations between number of mental disorders and past 30-day suicidal ideation (black) and past 30-days suicidal thoughts and behaviours (grey). Models adjusted for age group, sex, population group, and HIV status. Error bars indicate 95% confidence intervals (CIs). Suicidal ideation is a binary outcome modelled with logistic regression. Suicidal thoughts and behaviours are coded hierarchically (none, ideation, plan, attempt), with participants assigned to the highest level endorsed, and modelled with ordinal logistic regression.

**Figure S10: Associations between perceived stress and violence exposure and past 30-day suicidal thoughts and behaviours, adjusted for mental disorders**

Adjusted odds ratios (ORs) for associations between perceived stress, and violence experience and past 30-day suicidal ideation (black) and past 30-days suicidal thoughts and behaviours (grey). Models adjusted for age group, sex, population group, HIV status, and the presence of any current mental disorder. Error bars indicate 95% confidence intervals (CIs). Suicidal ideation is a binary outcome modelled with logistic regression. Suicidal thoughts and behaviours are coded hierarchically (none, ideation, plan, attempt), with participants assigned to the highest level endorsed, and modelled with ordinal logistic regression.

**Figure S11: Unadjusted odds ratios for factors associated with past 30-day suicidal thoughts and behaviours**

Unadjusted odds ratios (ORs) for factors associated with past 30-day suicidal ideation (black) and past 30-days suicidal thoughts and behaviours (grey). Error bars indicate 95% confidence intervals (CIs). Suicidal ideation is a binary outcome modelled with logistic regression. Suicidal thoughts and behaviours are coded hierarchically (none, ideation, plan, attempt), with participants assigned to the highest level endorsed, and modelled with ordinal logistic regression.
