# Supplementary material for: Suicidal thoughts and behaviours in Cape Town: A cross-sectional study of prevalence and social, contextual, and clinical correlates
Source: SSM Ment Health. Author manuscript; Available in PMC 2026 Aug 7. (PMC13441158; doi:10.1016/j.ssmmh.2026.100638)
Supplement: Supplementary file 3 [file NIHMS2197166-supplement-Table_S1.docx]

**Table S1: Prevalence of past 30-day suicidal thoughts and behaviours, hierarchically coded, by facility**

|  | **Facility 1** | **Facility 2** | **Facility 3** | **Total** |
| --- | --- | --- | --- | --- |
|  | N=401 | N=201 | N=11 | N=613 |
| Past 30-day suicidal thoughts and behaviours |  |  |  |  |
| None | 349 (87.0%) | 171 (85.1%) | 7 (63.6%) | 527 (86.0%) |
| Suicidal ideation | 45 (11.2%) | 15 (7.5%) | 3 (27.3%) | 63 (10.3%) |
| Suicidal plan | 3 (0.7%) | 5 (2.5%) | 1 (9.1%) | 9 (1.5%) |
| Suicide attempt | 4 (1.0%) | 10 (5.0%) | 0 (0.0%) | 14 (2.3%) |

Data are number of participants and (percentages).
